# “Improvements in Reflective Functioning in Patients with Treatment-Resistant Depression and Personality Disorder Treated with Esketamine”

**DOI:** 10.1192/j.eurpsy.2025.668

**Published:** 2025-08-26

**Authors:** M. Olivola, F. Mazzoni, G. Alessandro, A. Silva, N. Brondino, F. Calorio

**Affiliations:** 1 Department of mental health, ASST FBF SACCO, Milano; 2 DEPARTMENT OF BRAIN AND BEHAVIOURAL SCIENCE, ASST PAVIA; 3 DEPARTMENT OF BRAIN AND BEHAVIOURAL SCIENCE, University of Pavia, PAVIA; 4 Department of mental health, ASST PAVIA, Voghera, Italy

## Abstract

**Introduction:**

Reflective functioning, the cognitive ability to interpret both one’s own and others’ behaviors in terms of underlying mental states such as emotions, desires, intentions, and beliefs, is essential for effective social interaction, emotional regulation, and self-awareness and is compromised more in Personality disorders. In the context of depression, reflective functioning can be particularly compromised, leading to difficulties in processing emotional experiences and managing social interactions. (Williams, 2010; Luyten, 2013)

**Objectives:**

This study aims to understand the impact of esketamine on RF.

**Methods:**

Tool test: Montgomery-Åsberg Depression Rating Scale (MADRS) and the Reflective Functioning Questionnaire (RFQ-8), to monitor changes in depressive symptoms and mentalization ability across six stages of treatment with Spravato: before treatment (T0), after 1 week (T1), 1 month (T2), 2 months (T3), 3 months (T4), and 6 months (T5) of Spravato treatment.

**Results:**

The analysis revealed significant positive correlations between mentalization scores and the severity of depressive symptoms (MADRS):

- **T1 Mentalization and T1 MADRS:** The Pearson correlation coefficient of 0.349 indicates a moderate positive relationship, suggesting that poorer mentalization at Time 1 (T1) is associated with more severe depressive symptoms at the same time point.
- **T0 Mentalization and T3 MADRS:** The Pearson correlation coefficient of 0.392 shows a moderate positive association between mentalization at Time 0 (T0) and depressive symptoms at Time 3 (T3), indicating that poorer mentalization at T0 is related to more severe depressive symptoms later.
- **T2 Mentalization and T3 MADRS:** A Pearson correlation coefficient of 0.384 indicates a moderate positive relationship between mentalization at Time 2 (T2) and depressive symptoms at Time 3 (T3), suggesting that lower mentalization at T2 correlates with higher depressive symptoms at T3.

**Image 1:**

**Figure:**
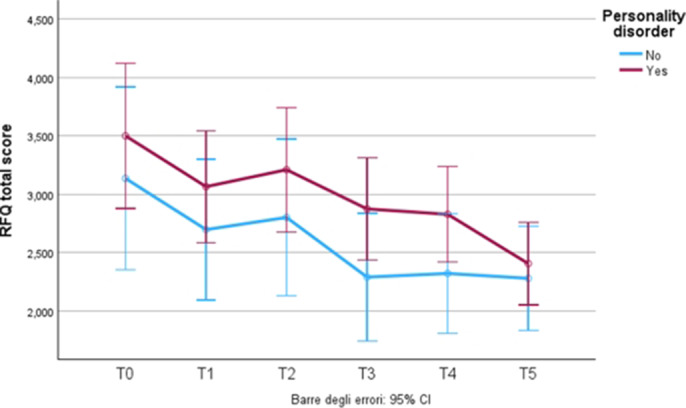

**Image 2:**

**Figure:**
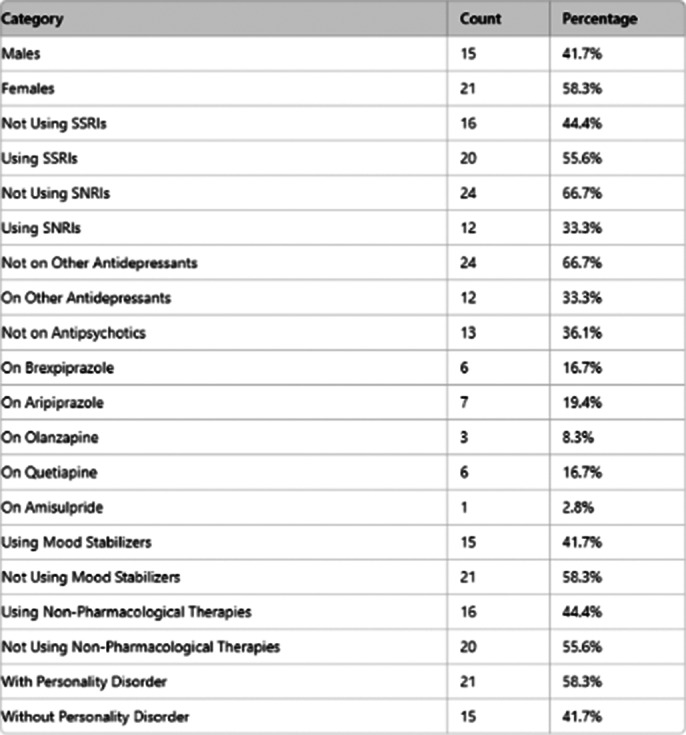

**Conclusions:**

The study assessed the effects of esketamine on depressive symptoms and reflective functioning (RF) in patients with treatment-resistant depression (TRD) and a 58% comorbidity rate of personality disorders. These findings underscore the potential impact of mentalization on the severity and trajectory of depressive symptoms. The observed correlations indicate that patients with mentalization scores above 3.5 generally report higher levels of depressive symptoms. These results highlight the significant role of mentalization in influencing the severity and progression of depressive symptoms.

**Disclosure of Interest:**

None Declared

